# Perioperative Outcomes in Antegrade, Retrograde, and Extracapsular Approaches to Parotidectomy in Benign and Malignant Neoplasms

**DOI:** 10.1002/ohn.70112

**Published:** 2026-01-08

**Authors:** Andrew D. P. Prince, Christian Schaaff, David Forner, Kimberly Oslin, Josh Smith, Michael M. Alevato, Emma Hershey, Lisa Chionis, Molly E. H. Neal, Mark E. P. Prince, Steven B. Chinn

**Affiliations:** ^1^ Department of Otolaryngology–Head and Neck Surgery University of Michigan Medical School Ann Arbor Michigan USA; ^2^ Now at Department of Otolaryngology–Head and Neck Surgery Henry Ford Hospital Detroit Michigan USA; ^3^ Now at Department of Otolaryngology–Head and Neck Surgery University of Pittsburgh Medical Center Pittsburgh Pennsylvania USA; ^4^ Now at Section of Otolaryngology–Head and Neck Surgery Dartmouth Hitchcock Medical Center Lebanon New Hampshire USA; ^5^ Now at Department of Otolaryngology–Head and Neck Surgery Kaiser Permanente Oakland Medical Center California Oakland USA

**Keywords:** head and neck, parotidectomy, salivary malignancy, salivary tumors, surgical approach

## Abstract

**Objective:**

To compare facial nerve function and perioperative outcomes among antegrade, retrograde, and extracapsular parotidectomy approaches in benign and malignant tumors.

**Study Design:**

Retrospective review.

**Setting:**

Tertiary academic center.

**Methods:**

Adults undergoing parotidectomy with facial nerve preservation between 2018 and 2023 were included. Demographic, surgical, and outcome variables were analyzed using bivariate and multivariable regression.

**Results:**

A total of 740 tumors were analyzed (353 benign, 387 malignant). Antegrade dissection was performed in 288 cases (194 malignant), retrograde in 334 (167 malignant), and extracapsular in 118 (26 malignant). Transient and permanent facial weakness occurred in 20% and 5% of cases. After adjustment, extracapsular dissection (odds ratio [OR] = 0.2, 95% confidence interval [CI] 0.1‐0.7, *P* = .0058) significantly reduced transient weakness compared with antegrade dissection. Retrograde dissection (OR = 0.5, 95% CI 0.3‐1.1, *P* = .11) demonstrated a trend toward lower rates of transient weakness relative to antegrade dissection. In malignant tumors, outcomes were similar across approaches. Extracapsular dissection was rarely used in malignant disease and reserved for selected cases. Antegrade dissection had longer operative times in benign and malignant tumors (*P* < .001). Perioperative complication rates, unplanned visits, recurrence, re‐operations, ED visits, and readmissions were low and not significantly different across techniques.

**Conclusion:**

In benign parotidectomy, extracapsular dissection significantly reduced transient facial weakness, while retrograde dissection demonstrated a trend toward reduced transient weakness compared with antegrade. In malignant disease, outcomes were similar across approaches. Selective application of extracapsular and retrograde techniques may optimize facial nerve outcomes and operative efficiency.

Salivary gland tumors account for 3% to 6% of head and neck neoplasms, with approximately 80% arising in the superficial lobe of the parotid gland and the majority being benign.^1^ Parotidectomy involves resecting the parotid gland to remove neoplastic tissue en bloc, with the goal of preserving facial nerve function when oncologically appropriate and minimizing procedure‐specific complications, such as Frey's syndrome, sialocele, and salivary fistula.^2^, ^3^

There are 3 primary surgical approaches to performing superficial parotidectomy.^4^ Antegrade dissection, the most traditional approach, consists of identifying the main nerve trunk as it emerges from the stylomastoid foramen and dissecting the branches within the parotid gland.^5^ Retrograde dissection involves identifying the distal branches of the facial nerve and tracing them proximally toward the main trunk.^5^, ^6^ Extracapsular dissection (ECD) involves dissection around the tumor capsule with preservation of nerve branches only if directly encountered.^7^, ^8^

Multiple studies have attempted to determine which approach is most effective and safe, but they have notable limitations. Prior studies assessing ECD have suggested comparable or reduced rates of facial nerve injury and postoperative complications to superficial parotidectomy; however, most have focused solely on benign tumors or grouped benign and malignant cases together without stratified analysis.^8^, ^9^, ^10^, ^11^, ^12^, ^13^, ^14^, ^15^ Additionally, studies have failed to distinguish between antegrade and retrograde techniques when evaluating superficial parotidectomy compared to ECD.^8^, ^9^, ^10^, ^11^, ^12^, ^13^, ^14^, ^15^, ^16^, ^17^ Comparative studies of antegrade versus retrograde have also yielded inconsistent findings, with most reporting no significant differences in facial nerve outcomes or complications.^5^, ^16^, ^17^ Few studies assess how surgical approach may affect other critical outcomes such as operative time, margin clearance, or complication rates like seroma or salivary fistula. Selecting the optimal approach remains uncertain and requires balancing oncologic principles, surgical access, and preservation of function.

To address these gaps, we directly compare all 3 approaches while accounting for both benign and malignant pathology. Our primary aim is to compare the incidence of postoperative facial nerve injury across these 3 techniques. Secondarily, we compare other relevant outcomes including operative time, recurrence, and common postoperative complications.

## Methods

### Study Design and Patient Population

This single‐center retrospective cohort study was conducted at a tertiary academic center and received exemption from the University of Michigan IRB Review Board (HUM00223009). We included patients ≥18 years who underwent parotidectomy with facial nerve preservation between 2018 and 2023. Benign tumor cases involving free flap or neck dissection were excluded; these were included for malignant tumors due to procedural relevance. Patients with preoperative facial nerve weakness, intraoperative nerve sacrifice for tumor invasion, prior parotid surgery, or previous radiation to the parotid were excluded from facial nerve outcome analyses to minimize confounding and isolate the effects of dissection technique on new postoperative weakness. These patients were retained in the overall cohort for descriptive analyses where applicable.

### Definitions and Data Collection

Demographics, Charlson Comorbidity Index (CCI), and procedure‐specific factors were collected. The choice of dissection approach was made by the operating surgeon based on preoperative assessment and intraoperative findings after flap elevation, including tumor size, location, and proximity to facial nerve branches. Facial nerve function was assessed postoperatively based on clinical documentation. Transient dysfunction was defined as any new postoperative weakness (complete or partial) that resolved by the last available follow‐up, and permanent dysfunction was defined as weakness persisting at or beyond 12 months of follow‐up, consistent with prior literature on facial nerve recovery after parotidectomy.^18^, ^19^ Cases with less than 12 months of follow‐up were categorized as transient to avoid overestimating rates of permanent dysfunction. The extracapsular approach for malignant tumors was performed in specific circumstances: preoperatively benign biopsy, highly superficial tumors, or known facial nerve invasion with preoperative paralysis. Dissection techniques (antegrade, retrograde, ECD) were categorized by facial nerve identification and relation to the tumor capsule. Total parotidectomy was defined as resection of tumor with deep parotid lobe extension. Approach to intraoperative facial nerve monitoring (via Medtronic NIM^TM^ or audiology) and drain placement were at surgeon discretion. Tumor size and AJCC 8th Edition tumor stage was gathered. Perioperative complications are defined as one or more complications within 30 days at the parotidectomy site, including hematoma, seroma, sialocele, infection, skin flap necrosis, salivary fistula, auricular nerve injury, Frey's syndrome, and medical complications. We also collected 30‐day postoperative ED visits, unplanned clinic visits, readmissions, and patient‐initiated communications. Re‐operation and tumor recurrence were tracked through final follow‐up.

### Statistical Analysis

Bivariate analyses comparing antegrade, retrograde, and extracapsular surgical approaches were conducted with two‐tailed analysis of variance for continuous measures and Chi‐Square for discrete measures to determine whether demographics, past medical history, intraoperative surgical factors, or subsequent complications were associated with surgical approach. Odds ratios were calculated for all discrete factor comparisons and Cramér's *V* (CV) statistics were calculated to assess degree of correlation.

Binary logistic regression adjusted for confounding factors when evaluating primary outcomes and secondary outcomes. Least squares regression models analyzed continuous variables. Variable selection was performed using backward selection on variables for which *P* < .25 in the bivariate analyses and subsequent forward selection, in accordance with the approach described in Hosmer et al.^20^ Clinically relevant factors, such as tumor size, tumor staging, concurrent procedures, CCI, surgeon, among others were specially considered. Assumptions were verified including the proportional hazards assumption, absence of multicollinearity, and the Hosmer‐Lemeshow goodness of fit. A significance level of *P* < .05 was used. All analyses were performed using JMP (Version 17) Statistical Software.

## Results

### Patient Characteristics

Of 740 total tumors selected for analysis, 353 were benign and 387 were malignant. For benign tumors, 94 (27%) underwent antegrade 167 (47%) retrograde, and 92 (26%) extracapsular approaches (Table 1A). For malignant tumors, the antegrade approach was used in 194 (50%) patients, the retrograde approach in 167 (43%) patients, and the extracapsular approach in 26 (7%) patients (Table 1B). Overall, 42% of patients were females. Age, sex, BMI, alcohol use, tobacco use, and CCI did not show significant differences across the 3 surgical approaches for either benign or malignant tumors.

**Table 1A:** Benign Tumor Patient Characteristics

Patient characteristics	Antegrade (n = 94)	Retrograde (n = 167)	Extracapsular (n = 92)	*P*‐value	Cramer's *V*
Sex (F/M)	51/43 (54)	97/70 (58)	52/40 (56)	.8355	0.03
Caucasian (n, (%))	72 (77)	144 (86)	75 (82)	.1447	0.11
African American (n, (%))	4 (4)	14 (8)	6 (7)	.4215	0.07
American Indian (n, (%))	1 (1)	3 (2)	0 (0)	.2648	0.07
Asian (n, (%))	6 (6)	2 (1)	4 (4)	.0628	0.12
Other race (n, (%))	10 (11)	2 (1)	2 (2)	.0014	0.21
Hispanic/Latino (n, (%))	8 (9)	4 (2)	0 (0)	.0022	0.18
Unknown ethnicity (n, (%))	2 (2)	8 (5)	2 (2)	.3889	0.07
Charlson Comorbidity Index >1 (n, (%))	47 (50)	99 (59)	51 (55)	.3492	0.05
Tobacco use (n, (%))	46 (49)	85 (51)	43 (47)	.8117	0.03
Alcohol use (n, (%))	39 (41)	70 (42)	46 (50)	.3925	0.07
Age (mean [95% CI])	53.5 (50.5, 56.6)	56.8 (54.5, 59.1)	55.2 (52.1, 58.4)	.2534	‐
BMI (mean [95% CI])	29.8 (28.3, 31.3)	31.3 (30.2, 32.4)	31.1 (29.6, 32.6)	.2400	‐

Abbreviations: BMI, body mass index; CI, confidence interval.

**Table 1B:** Malignant Tumor Patient Characteristics

Patient characteristics	Antegrade (n = 194)	Retrograde (n = 167)	Extracapsular (n = 26)	*P*‐value	Cramer's *V*
Sex (F/M) (n, (%))	49/145 (25)	48/119 (29)	15/11 (42)	.2002	0.09
Caucasian (n, (%))	178 (92)	153 (92)	25 (96)	.6730	0.04
African American (n, (%))	7 (4)	5 (3)	0 (0)	.4061	0.05
American Indian (n, (%))	2 (1)	0 (0)	0 (0)	.2500	0.07
Asian (n, (%))	3 (2)	3 (2)	0 (0)	.6455	0.04
Other race (n, (%))	2 (1)	1 (1)	0 (0)	.7307	0.03
Hispanic/Latino (n, (%))	0 (0)	1 (1)	0 (0)	.4308	0.06
Unknown ethnicity (n, (%))	7 (4)	6 (4)	1 (4)	.9979	0.00
Charlson Comorbidity Index > 1 (n, (%))	189 (97)	156 (93)	24 (92)	.1393	0.10
Tobacco use (n, (%))	102 (53)	84 (51)	11 (42)	.5890	0.05
Alcohol use (n, (%))	81 (42)	60 (38)	14 (54)	.3197	0.08
Age (mean (95% CI))	68.6 (66.5, 70.8)	68.7 (66.4, 71.0)	68.9 (63.0, 74.7)	.9972	‐
BMI (mean (95% CI))	29.1 (28.2, 30.1)	29.5 (28.5, 30.5)	26.8 (24.1, 29.4)	.1679	‐

Abbreviations: BMI, body mass index; CI, confidence interval.

### Surgical Characteristics

Among benign tumors, 82% of extracapsular, 62% of retrograde, and only 36% of antegrade dissections were performed outpatient (*P* < .0001, CV = 0.34, Table 2A). A similar pattern was observed in malignant tumors, with 54% of extracapsular, 16% of retrograde, and 7% of antegrade cases performed in the outpatient setting (*P* < 0.0001, CV = 0.33, Table 2B).

**Table 2A:** Benign Tumors Surgical Characteristics

Surgical characteristics	Antegrade (n = 94)	Retrograde (n = 167)	Extracapsular (n = 92)	*P*‐value	Cramer's *V*
Out‐patient setting (n, (%))	34 (36)	103 (62)	75 (82)	<.0001	0.34
Prior parotid surgery (n, (%))	14 (15)	12 (7)	12 (13)	.1010	0.11
Prior radiation to parotid (n, (%))	0 (0)	0 (0)	0 (0)	‐	‐
Preoperative facial nerve dysfunction (n, (%))	0 (0)	0 (0)	0 (0)	‐	‐
Facial nerve monitoring (n, (%))	90 (96)	165 (99)	83 (90)	.0055	0.17
Concurrent procedure (n, (%))	15 (16)	23 (14)	14 (15)	.9064	0.02
Drain (n, (%))	52 (55)	53 (32)	13 (14)	<.0001	0.32
Total parotidectomy (n, (%))	32 (34)	65 (39)	39 (42)	.4976	0.06
Tumor size (cm, (SD))	2.5 (2.2, 2.8)	2.8 (2.6, 3.1)	2.6 (2.3, 2.9)	.1926	‐

**Table 2B:** Malignant Tumor Surgical Characteristics

Surgical characteristics	Antegrade (n = 194)	Retrograde (n = 167)	Extracapsular (n = 26)	*P*‐value	Cramer's *V*
Out‐patient setting (n, (%))	14 (7)	27 (16)	14 (54)	<.0001	0.33
Prior parotid surgery (n, (%))	9 (5)	13 (8)	3 (12)	.2914	0.08
Prior radiation to parotid (n, (%))	4 (2)	6 (4)	1 (4)	.6464	0.05
Preoperative facial nerve dysfunction (n, (%))	5 (3)	8 (5)	3 (12)	.1355	0.29
Facial nerve monitoring (n, (%))	183 (94)	146 (88)	18 (69)	.0009	0.21
Concurrent procedure (n, (%))	170 (89)	135 (84)	19 (73)	.1125	0.11
Drain (n, (%))	169 (88)	132 (80)	9 (35)	<.0001	0.33
Total parotidectomy (n, (%))	72 (37)	52 (31)	8 (31)	.4571	0.06
Free flap used (n, (%))	33 (17)	54 (33)	5 (19)	.0025	0.18
Neck dissection (n, (%))	145 (75)	103 (62)	5 (19)	<.0001	0.29
Tumor metastatic to parotid (n, (%))	93 (48)	81 (49)	9 (35)	.3928	0.07
T Stage >2 (n, (%))	53 (28)	52 (32)	7 (29)	.6763	0.05
N Stage >0 (n, (%))	123 (64)	79 (49)	7 (32)	.0012	0.19
M Stage >0 (n, (%))	5 (3)	4 (3)	1 (5)	.8778	0.03
Tumor size (cm, (SD))	2.9 (2.5, 3.2)	3.0 (2.6, 3.3)	2.4 (1.6, 3.2)	.4983	‐

Total parotidectomies (n = 268) were performed for both benign (n = 136, 38.5%) and malignant tumors (n = 132, 34.1%) without significant differences in choice of approach. Concurrent procedures, such as neck dissection, free flap, wide local excision, auriculectomy, and so forth, were also performed in malignant tumors without significant differences in approach. Facial nerve monitoring was used for most cases. The exceptions were for some very low or superficial tumors, unanticipated parotid tissue resection, or cases involving facial nerve sacrifice. Of the benign tumors resected with facial nerve monitoring (n = 341), 48.9% of antegrade, 49.1% of retrograde, and 54.1% of extracapsular dissections were performed with NIMS rather than monitoring by trained audiologists (*P* = .7174). For malignant tumors, 38.3% of antegrade, 58.9% of retrograde, and 44.4% of extracapsular dissections were performed via NIMs rather than via monitoring by trained audiologists (*P* = .0009). In benign tumors, drain placement was more common after the antegrade approach (55%) followed by retrograde (32%) then extracapsular approach (14%) (*P* < .0001, CV = 0.32). This trend also held for malignant tumors, with drain placement more commonly employed after the antegrade (88%) and retrograde (80%) approaches than the extracapsular approach (35%) (*P* < .0001, CV = 0.33).

For malignant tumors, less than 30% of tumors were T stage >2, about half were N > 0 stage, and only 10 were metastatic. Neck dissection was performed in 75% of antegrade, 62% of retrograde, and 19% of extracapsular dissections (*P* < .0001, CV  = 0.18). Similarly, free flap reconstruction was used in 33% of retrograde cases, compared to 17% of antegrade and 19% of extracapsular approaches (*P* = .0019, CV =  0.14).

### Tumor Characteristics

For benign tumors, there were 183 (52%) pleomorphic adenomas, with no significant differences in their distribution across surgical approach (*P* = .2993, Table 3A). Warthin tumors (n = 60; 17%) were similarly distributed across approaches with no significant differences (*P* = .7521). The same held for other benign tumors (*P* = .6437). 70 cases with preoperative biopsies that were nondiagnostic or concerning for neoplasm were found to have no tumor and be benign gland, fibrosis, or cystic on final pathology.

**Table 3A:** Benign Tumor Types

Benign tumor types	Antegrade (n = 94)	Retrograde (n = 167)	Extracapsular (n = 92)	*P*‐value	Cramer's *V*
Pleomorphic adenoma (n, (%))	43 (46)	93 (56)	47 (51)	.2993	0.08
Warthin tumor (n, (%))	15 (16)	27 (16)	18 (20)	.7521	0.04
Benign parotid tissue (n, (%))	27 (29)	27 (16)	16 (17)	.0476	0.13
Other (n, (%))	9 (10)	20 (12)	11 (12)	.6437	0.05

For malignant neoplasms, squamous cell carcinoma (n = 140) and melanoma (n = 53) were the most common tumor types excised (Table 3B). Melanoma accounted for 18% of antegrade, 11% of retrograde, and 3% of extracapsular dissections (*P* = .0085). Adenoid cystic carcinoma (=10) was notable for a complete absence in the antegrade approach but was present in the retrograde (5%) and extracapsular (6%) approaches (*P* = .0009). Merkel cell carcinoma (n = 19) was also observed more frequently in the antegrade approach (7%) compared to the retrograde (3%) and was absent in the extracapsular approach (*P* = .0346).

**Table 3B:** Malignant Tumor Types

Malignant tumor types	Antegrade (n = 194)	Retrograde (n = 167)	Extracapsular (n = 26)	*P*‐value	Cramer's *V*
Acinar cell carcinoma (n, (%))	10 (5)	6 (4)	0 (0)	.2468	0.07
Adenoid cystic carcinoma (n, (%))	0 (0)	8 (5)	2 (8)	.0007	0.17
Basal cell carcinoma (n, (%))	6 (3)	5 (3)	6 (23)	.0015	0.24
Sarcoma (n, (%))	7 (4)	6 (4)	1 (4)	.9979	0.00
Melanoma (n, (%))	35 (18)	17 (10)	1 (4)	.0222	0.13
Merkel cell carcinoma (n, (%))	14 (7)	5 (3)	0 (0)	.0479	0.11
Mucoepidermoid carcinoma (n, (%))	8 (4)	18 (11)	2 (8)	.0491	0.12
Salivary duct carcinoma (n, (%))	7 (4)	6 (4)	1 (4)	.9979	0.00
Squamous cell carcinoma (n, (%))	62 (32)	70 (42)	8 (31)	.1228	0.10
Other (n, (%))	45 (23)	26 (16)	5 (19)	.1874	0.09

### Primary Objective

For benign tumors, 313 met criteria for facial weakness analysis and 55 patients had either temporary or permanent weakness. There were 4 permanently weak nerves, 25% involved all branches (House Brackman 3), the rest were the marginal mandibular. For the transient weakness, 65% were the marginal mandibular, 13% upper division, 10% the lower division, 7% all branches, 5% a combination of 1 upper and 1 lower branch.

Significant findings in benign tumor excision included an increased incidence of unintentional facial nerve injury in the antegrade approach (3% [*P* = .0452, CV = 0.12)] (Table 4). In benign tumors, transient facial nerve weakness was more frequent in the antegrade approach (24%) compared to retrograde (14%) and extracapsular (8%) approaches (*P* = .0159, CV = 0.15).

**Table 4:** Benign Tumor Outcomes

Outcome measures: benign tumors	Antegrade (n = 94)	Retrograde (n = 167)	Extracapsular (n = 92)	*P*‐value	Cramer's *V*
Peri‐operative complication (n, (%))	29 (31)	36 (22)	17 (18)	.1311	0.11
Hematoma (n, (%))	1 (1)	4 (2)	1 (1)	.6287	0.05
Seroma (n, (%))	3 (3)	10 (6)	4 (4)	.5704	0.06
Sialocele (n, (%))	5 (5)	11 (7)	2 (2)	.2508	0.08
Surgical site infection (n, (%))	4 (4)	3 (2)	3 (3)	.4951	0.06
Skin flap necrosis (n, (%))	1 (1)	0 (0)	0 (0)	.2652	0.09
Salivary fistula (n, (%))	2 (2)	0 (0)	1 (1)	.1230	0.10
Unintentional facial nerve injury (n, (%))	3 (3)	0 (0)	1 (1)	.0452	0.12
Purposeful facial nerve resection (n, (%))	0 (0)	3 (2)	1 (1)	.2591	0.07
Temp. facial nerve weakness (n, (%))	19 (24)	22 (14)	6 (8)	.0159	0.15
Permanent facial nerve injury (n, (%))	2 (3)	1 (1)	1 (1)	.5177	0.07
Great auricular nerve injury (n, (%))	7 (7)	9 (5)	3 (3)	.4389	0.07
Scarring (n, (%))	2 (2)	5 (3)	3 (3)	.8783	0.03
Frey syndrome (n, (%))	1 (1)	1 (1)	2 (2)	.5436	0.06
First bite syndrome (n, (%))	1 (1)	0 (0)	0 (0)	.2652	0.09
Other complications (n, (%))	2 (2)	1 (1)	1 (1)	.5556	0.06
Tumor recurrence (n, (%))	3 (3)	1 (1)	0 (0)	.0823	0.12
Reoperation (n, (%))	2 (2)	2 (2)	2 (2)	.7827	0.04
Readmission for surgical complication (n, (%))	0 (0)	1 (1)	0 (0)	.4723	0.04
ED visit for surgical complication (n, (%))	1 (1)	3 (2)	0 (0)	.2648	0.07
Unplanned clinic visit for surgical complication (n, (%))	6 (6)	10 (6)	3 (3)	.5403	0.06
Call to provider for surgical complication (n, (%))	38 (40)	68 (41)	25 (27)	.0661	0.12
Operative time (n, (%))	162.8 (151.7, 173.8)	103.6 (95.2, 111.9)	79.7 (68.6, 90.9)	<.0001	‐
Est. blood loss (mL, (SD))	35.6 (26.8, 44.5)	30.3 (23.5, 37.1)	15.9 (6.9, 24.8)	.0058	‐
Mean follow‐up time (months, (SD))	9.8 (6.4, 13.1)	7.4 (4.9, 10.0)	9.6 (6.2, 13.0)	.4584	‐

**Figure 1:**
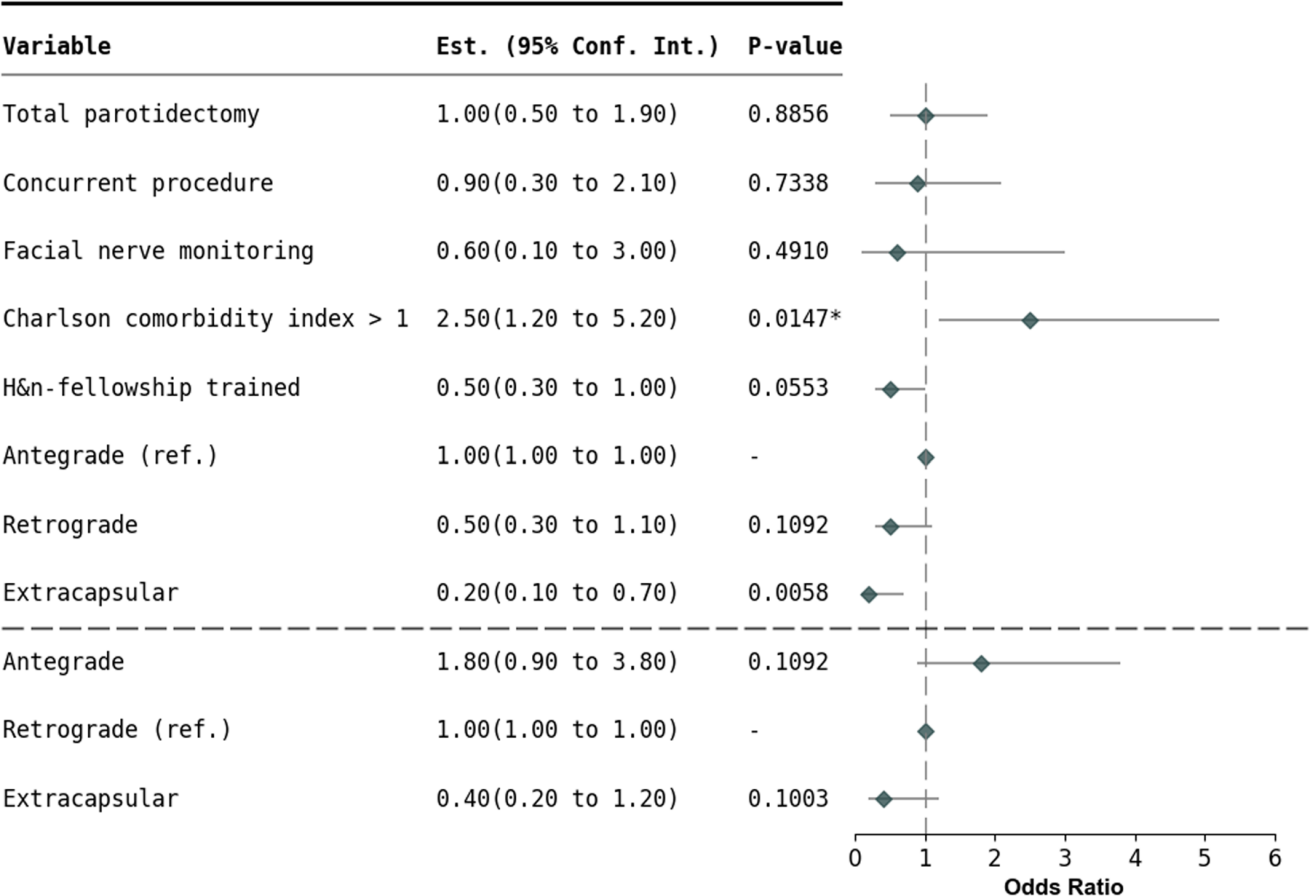
Logistic regression analysis of temporary facial nerve weakness in benign tumor resection.

After adjusting for potential confounding factors in logistic regression analysis, extracapsular dissection (OR = 0.2, 95% CI 0.1‐0.6, *P* = .0058) significantly reduced transient weakness compared with antegrade dissection (Figure 1). Retrograde dissection (OR = 0.5, 95% CI 0.3‐1.1, *P* = .11) demonstrated a trend toward lower rates of transient weakness relative to antegrade dissection (Figure 1).

For malignant tumors, 283 met criteria for facial nerve analysis and 119 had either temporary or permanent weakness. There were 44 permanently weak nerves, 25% involved all branches, 61% were the marginal mandibular, 14% were upper branches. For transient weakness, 72% were marginal mandibular, 8% were all branches, 11% upper division, 4% lower division, 5% a combination of 1 upper and 1 lower branch.

**Figure 2:**
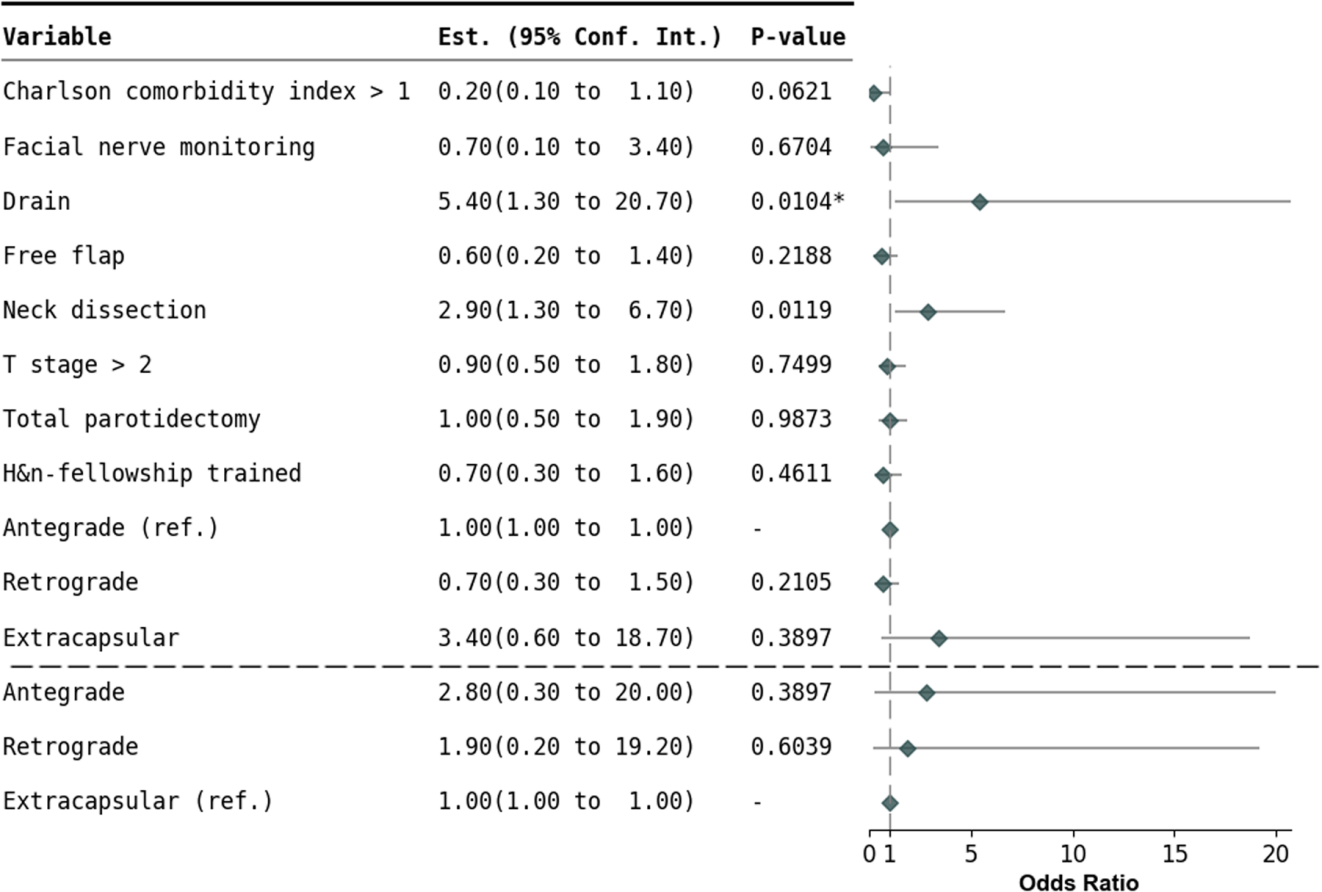
Logistic regression analysis of facial nerve weakness in malignant tumor resection.

In malignant tumors, purposeful facial nerve resection was most common in the retrograde approach (26% [*P* = .0255, CV = 0.14]) (Table 5). Transient facial nerve weakness was more frequent in the antegrade approach (34%) compared to retrograde (21%) and extracapsular (5%) approaches (*P* = .0021, CV = 0.21). After adjustment in malignant tumors, there were no significant differences in transient facial nerve weakness observed between surgical approaches (Figure 2). Additionally, after adjustment, there were no significant differences in permanent facial nerve injury across approaches for either benign or malignant tumors.

**Table 5:** Malignant Tumor Outcomes

Outcome measures: malignant tumors	Antegrade (n = 194)	Retrograde (n = 167)	Extracapsular (n = 26)	*P*‐value	Cramer's *V*
Peri‐operative complication (n, (%))	46 (24)	48 (29)	4 (15)	.2484	0.08
Hematoma (n, (%))	7 (4)	12 (7)	1 (4)	.2987	0.08
Seroma (n, (%))	9 (5)	12 (7)	1 (4)	.5340	0.06
Sialocele (n, (%))	2 (1)	0 (0)	0 (0)	.2500	0.07
Surgical site infection (n, (%))	10 (5)	8 (5)	0 (0)	.2723	0.06
Skin flap necrosis (n, (%))	4 (2)	4 (2)	1 (4)	.8663	0.03
Salivary fistula (n, (%))	1 (1)	0 (0)	0 (0)	.5006	0.05
Unintentional facial nerve injury (n, (%))	4 (2)	6 (4)	1 (4)	.6464	0.05
Purposeful facial nerve resection (n, (%))	30 (15)	43 (26)	2 (8)	.0102	0.15
Temp. facial nerve weakness (n, (%))	51 (34)	23 (21)	1 (5)	.0021	0.21
Permanent facial nerve injury (n, (%))	24 (16)	19 (17)	1 (5)	.3036	0.10
Great auricular nerve injury (n, (%))	8 (4)	7 (4)	0 (0)	.3446	0.05
Scarring (n, (%))	4 (2)	4 (2)	1 (4)	.8663	0.03
First bite syndrome (n, (%))	1 (1)	0 (0)	0 (0)	.5006	0.05
Trismus (n, (%))	2 (1)	1 (1)	0 (0)	.7307	0.03
Other complications (n, (%))	6 (3)	9 (5)	2 (8)	.4082	0.07
Tumor recurrence (n, (%))	50 (26)	28 (17)	6 (23)	.1115	0.11
Reoperation (n, (%))	12 (6)	9 (5)	3 (12)	.5457	0.06
Readmission for surgical complication (n, (%))	5 (3)	3 (2)	1 (4)	.7799	0.04
ED visit for surgical complication (n, (%))	6 (3)	8 (5)	2 (8)	.4914	0.06
Unplanned clinic visit for surgical complication (n, (%))	5 (3)	5 (3)	0 (0)	.4800	0.05
Call to provider for surgical complication (n, (%))	75 (39)	61 (37)	6 (23)	.2794	0.08
Operative time (min, (SD))	334.7 (313.4, 356.0)	265.5 (241.8, 289.1)	146.3 (86.1, 206.5)	<.0001	‐
Est. blood loss (mean, (SD)	148.5 (119.9, 177.0)	175.1 (144.2, 205.9)	65.0 (−12.0, 142.0)	.0291	‐
Mean follow‐up time (months, (SD))	20.3 (17.9, 22.8)	16.6 (14.0, 19.2)	15.5 (8.8, 22.2)	.0838	‐

### Secondary Objectives

**Figure 3:**
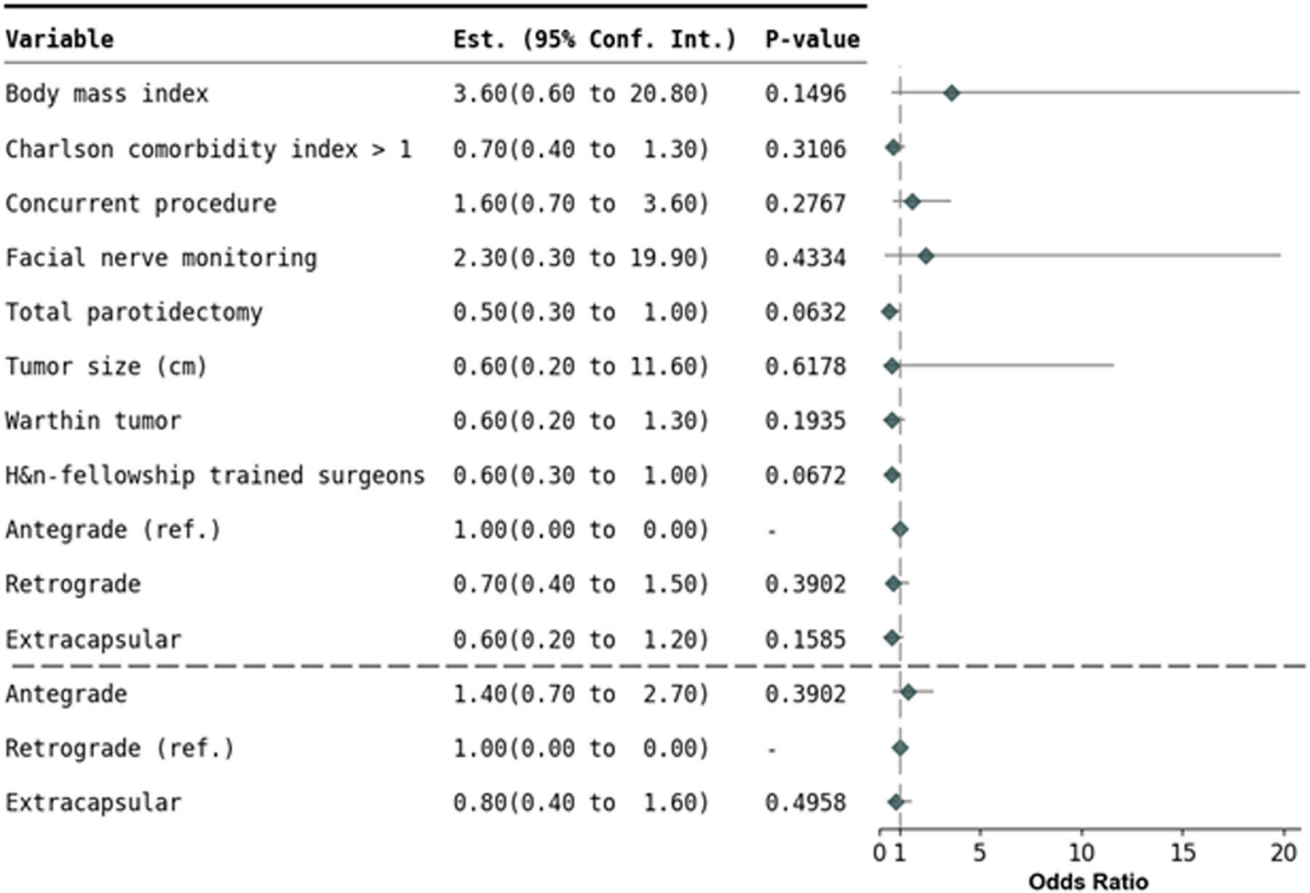
Logistic regression analysis of temporary perioperative complication rates in benign tumor resection.

**Figure 4:**
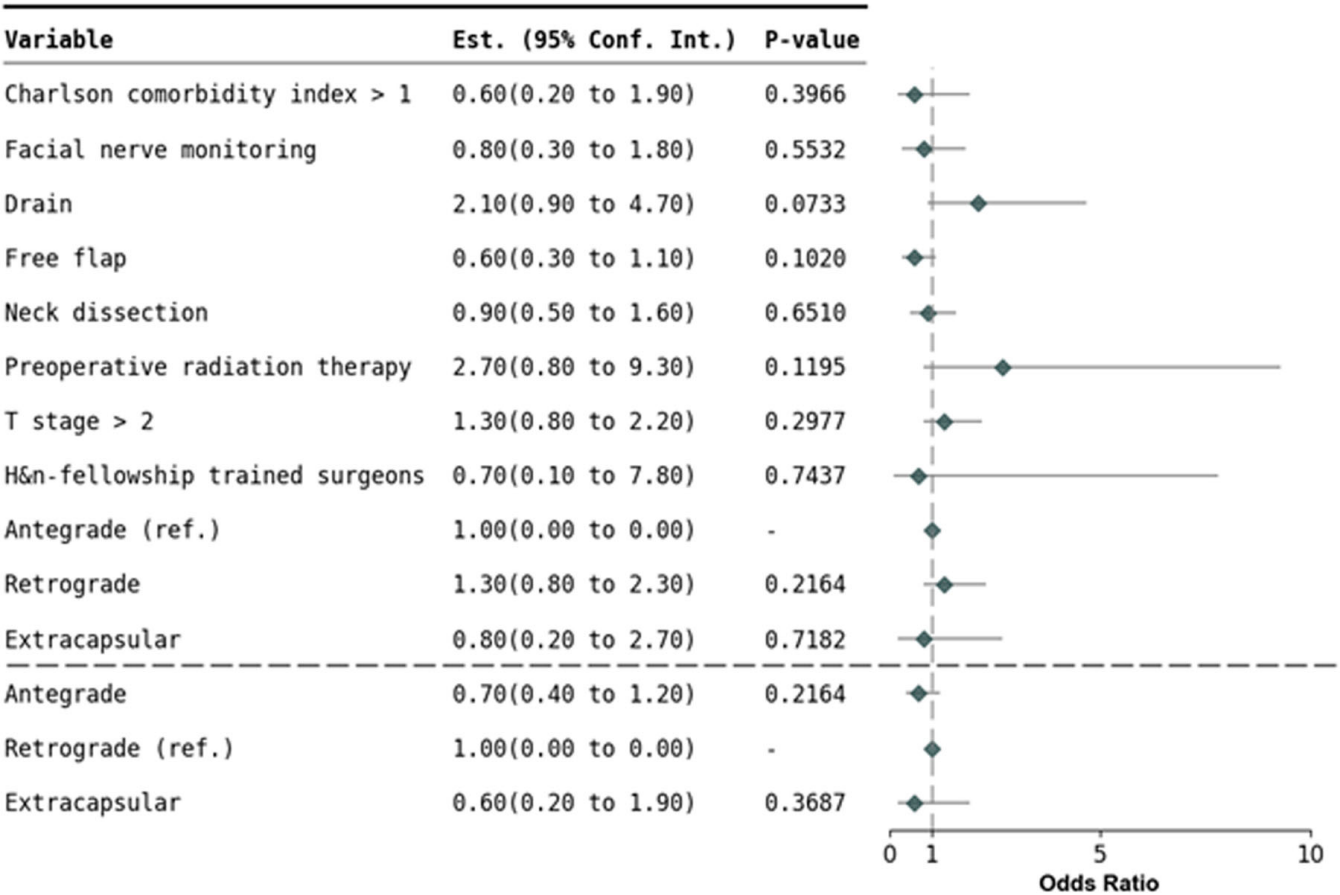
Logistic regression analysis of temporary perioperative complication rates in malignant tumor resection.

Overall mean follow‐up time was 8.6 months for benign and 18.4 months for malignant parotidectomy. Tumor size and mean follow‐up time did not show significant differences across the surgical approaches. After adjusting for potential confounding factors in logistic regression analysis, there were no significant differences in overall perioperative complications, including hematoma, seroma, sialocele, surgical site infection, skin flap necrosis, salivary fistula, Stensen duct injury, permanent facial nerve injury, scarring, Frey syndrome, first bite syndrome, trismus, and other complications in benign and malignant tumors (Figures 3 and 4). No significant differences between approaches for 30‐day readmission for surgical complication, emergency department visit for surgical complication, unplanned clinic visit for surgical complication, and call to provider for surgical complication were found (Tables 4 and 5). Similarly, there was no difference in tumor recurrence and re‐operation from initial surgery to the most recent follow‐up.

For benign tumors when accounting for possible confounding factors in logistic regression analysis, the extracapsular approach required shorter operative times than the retrograde approach (beta = −6.1, 95% CI: (−12.5, −0.2), *P* < .0001), and the retrograde approach required shorter operative times than the antegrade approach (beta = −21.7, 95% CI: (−28.4, −15.0), *P* < .001) (Supplemental Figure S1, available online). This trend was also observed in malignant tumors, the extracapsular approach was found to require less operative time than the retrograde approach, though this result was not statistically significant (beta = −6.1, 95% CI: (−12.5, −0.2), *P* = .0576), retrograde approach to parotidectomy was found to have significantly shorter operative time than the antegrade approach to malignant tumors (beta = −36.2, 95% CI: (−49.7, −23.5), *P* < .0001) (Supplemental Figure S2, available online).

We evaluated malignant primary parotid tumors (n = 206) versus metastatic tumors to the parotid gland (n = 188) (Supplemental Table S1, available online). Metastatic tumors were more likely to recur (26% vs 17%, *P* = .024), though there were no significant differences across approach, facial nerve weakness, perioperative complications, or any other outcome measured.

### Surgeon Specific Analysis

Most of the surgeries were performed by 8 fellowship‐trained head and neck surgeons (71% benign, 76.5% malignant). Other procedures were performed by general otolaryngologists, oral maxillofacial surgeons, and fellows. In benign tumors, 2 of the fellowship‐trained head and neck surgeons performed more retrograde dissections than other types, 1 performed more extracapsular, and one performed more antegrade (Supplemental Table S2A, available online). In malignant cases, there were no significant differences in the selected approach across surgeons, with low frequency of extracapsular dissection for any surgeon (Supplemental Table S2B, available online). In benign tumors, surgeon 4 had lower rates of temporary weakness but did not favor a specific approach (Table 6A). When combined, the fellowship‐trained surgeons had lower rates of temporary FN weakness than other surgeons (*P* = .0082) and perioperative complications (*P* = .0371) (Table 6A). After adjusting for confounders, only temporary facial nerve weakness in benign tumors was lower with fellowship‐trained surgeons (OR = 0.5; 95% CI: 0.3‐0.9; *P* = .0132). In malignant tumors, surgeon 5 had higher rates of temporary facial nerve weakness but did not favor a specific approach (Table 6B). Otherwise, there were no significant differences in temporary or permanent facial nerve weakness, or perioperative complications for malignant tumors (Table 6B).

**Table 6A:** Surgeon Specific Analysis for Benign Tumors

Surgeon‐specific analysis: Benign	Total cases included	Temp. facial nerve weakness	*P*‐value	Permanent facial nerve weakness	*P*‐value	Perioperative complications	*P*‐value
Surgeon 1 (n, (%))	0	0 (0)	‐	0 (0)	‐	0 (0)	.4651
Surgeon 2 (n, (%))	57	5 (10)	.0706	0 (0)	.2031	9 (14)	.0353
Surgeon 3 (n, (%))	48	2 (4)	.0049	1 (25)	.6143	7 (14)	.0642
Surgeon 4 (n, (%))	14	0 (0)	.0238*	0 (0)	.5438	3 (20)	.7478
Surgeon 5 (n, (%))	1	1 (100)	.0562	0 (0)	.8725	0 (0)	.4651
Surgeon 6 (n, (%))	33	9 (18)	.09	0 (0)	.3435	8 (24)	.9828
Surgeon 7 (n, (%))	23	5 (10)	.4788	0 (0)	.433	12 (38)	.0479
Surgeon 8 (n, (%))	51	7 (14)	.5803	1 (25)	.6546	13 (23)	.9745
Other surgeons (n, (%))	86	22 (43)	.0082*	2 (50)	.3374	31 (31)	.0371

**Table 6B:** Surgeon Specific Analysis for Malignant Tumors

Surgeon‐specific analysis: malignant	Total cases included	Temp. facial nerve weakness	*P*‐value	Permanent facial nerve weakness	*P*‐value	Perioperative complications	*P*‐value
Surgeon 1 (n, (%))	19	4 (5)	.5623	6 (14)	.0647	9 (35)	.3034
Surgeon 2 (n, (%))	26	4 (5)	.1529	4 (9)	.9937	10 (28)	.7801
Surgeon 3 (n, (%))	28	5 (7)	.2523	2 (5)	.1465	11 (20)	.314
Surgeon 4 (n, (%))	23	7 (9)	.6678	4 (9)	.8511	8 (24)	.747
Surgeon 5 (n, (%))	70	29 (39)	.0016*	13 (30)	.3952	17 (20)	.1211
Surgeon 6 (n, (%))	22	5 (7)	.6641	4 (9)	.8511	10 (28)	.7801
Surgeon 7 (n, (%))	8	1 (1)	.3240	3 (7)	.1199	3 (28)	.9118
Surgeon 8 (n, (%))	31	5 (7)	.1432	3 (7)	.3296	17 (38)	.0602
Other Surgeons (n, (%))	55	15 (20)	.8994	5 (11)	.1178	17 (26)	.9894

## Discussion

This study of 740 parotidectomy cases is the largest single‐center retrospective cohort to evaluate postoperative outcomes of parotidectomy by surgical approach: antegrade, retrograde, and extracapsular dissection, in both benign and malignant tumors. In benign tumors, extracapsular dissection was associated with significantly lower rates of transient facial nerve weakness, while retrograde dissection showed a trend toward reduced rates compared with antegrade, after adjustment for confounders. In malignant tumors, no differences in nerve outcomes were observed across approaches.

Our findings are supported by prior evidence suggesting reduced rates of transient postoperative facial nerve paralysis with extracapsular dissection relative to superficial dissection.^2^, ^8^, ^10^, ^11^, ^12^, ^13^, ^15^ The only systematic review evaluating antegrade vs retrograde found no significant difference in temporary or permanent facial nerve injury with an almost equal number of patients to our series.^21^ However, prior work from our institution showed retrograde dissection of the facial nerve for benign parotid tumors is associated with reduced operative times, postoperative admission rates, and rates of facial nerve paresis compared to antegrade even when controlling for tumor location.^22^ Our study is the first to compare retrograde and extracapsular approaches, with a trend toward reduced transient facial nerve weakness in extracapsular cases. While not fully assessed in this study, our surgeons often selected extracapsular dissection for more superficial and tail of the parotid tumors. These findings support the utility of retrograde and extracapsular approaches in preserving facial nerve function, with the retrograde offering potentially more versatility for complex tumors.

In malignant tumors, no significant differences in transient or permanent facial nerve weakness were observed among approaches following adjustment for tumor complexity indicators such as neck dissection and free flap reconstruction. While we included malignant cases excised with an extracapsular approach in this analysis, it is important to recognize they were performed under special circumstances (preoperatively benign, highly superficial tumors, or known facial nerve invasion), which may introduce selection bias. As anticipated, malignant tumors exhibited higher rates of permanent facial nerve injury, but this was not impacted by surgical approach. This suggests that tumor biology and proximity to the facial nerve may have greater effect than dissection technique.

In both malignant and benign cases, perioperative complication rates (eg, Frey's syndrome, hematoma formation, etc.) were low with no statistically significant differences found among the approaches. We found that antegrade and retrograde approaches resulted in slightly higher overall perioperative complication rates than extracapsular dissection, though these results were not significant after adjustment. Most studies show few differences in postoperative complication rates between dissection techniques, but some studies suggest that extracapsular dissection results in lower rates of Frey's syndrome, hematoma formation, and higher rates of capsular rupture than superficial parotidectomy.^5^, ^12^, ^16^, ^17^, ^19^, ^23^, ^24^, ^25^ However, our data suggest that surgical approach has limited impact on complication incidence when accounting for confounders. This is further supported by rates of 30‐day postoperative communication to providers, emergency department visits, unplanned clinic visits, and readmission which were not significantly different.

As in previous studies, recurrence rates of benign tumors were low (3 in antegrade, 1 in retrograde, and 0 in extracapsular), limiting comparative analysis.^5^, ^16^, ^19^, ^26^, ^27^ Malignant tumors were more complex with higher rates of morbidity and recurrence, but there were no significant differences by approach. A comprehensive evaluation of recurrence was outside the scope of this study as we did not stratify by local versus distant recurrence, tumor stage, and survival outcomes, which warrants further investigation.

In both benign and malignant tumors, operative time was the shortest in extracapsular dissection followed by retrograde, then antegrade. This supports the selective use of extracapsular and retrograde approaches to improve efficiency in appropriate cases. We also found, in benign tumors, that after adjustment, high‐volume fellowship‐trained surgeons had lower rates of temporary facial nerve weakness; outcomes did not differ among these fellowship‐trained surgeons despite different approach preferences.

This study is limited by its retrospective design, variable follow‐up, and single‐center scope. Approach selection inherently reflects surgeon preference and intraoperative judgment, as anatomic variations and tumour‐to‐nerve relationships are not always apparent preoperatively or on imaging. Because these anatomic relationships are often best appreciated intraoperatively, no standardized imaging‐based criteria were applied. Patients with follow‐up shorter than 12 months were conservatively classified as having transient weakness, consistent with literature indicating most recovery occurs within 1 year; this may slightly underestimate permanent dysfunction. Patients with preoperative facial nerve weakness, prior radiation, or previous parotid surgery were excluded from facial nerve outcome analysis to reduce confounding from preexisting or treatment‐related nerve dysfunction. While this approach strengthens internal validity by isolating the effect of surgical technique, it may limit generalizability to more complex or previously treated cases. Some postoperative complications treated outside our system may have been missed, and temporal changes, such as increased outpatient surgery during the COVID‐19 pandemic, could introduce bias.

## Conclusion

In this large, single‐center retrospective cohort, extracapsular dissection significantly reduced transient facial nerve weakness in benign parotid tumors, while retrograde dissection demonstrated a trend toward lower rates compared with the traditional antegrade approach. Both extracapsular and retrograde approaches were associated with shorter operative times in benign cases. In malignant tumors, facial nerve outcomes were similar across techniques, likely due to greater tumor complexity and oncologic demands. Extracapsular dissection was rarely used in malignant tumors and only in highly selective situations, limiting definitive conclusions in this subgroup. Overall complication and recurrence rates were low and did not significantly differ by approach. These findings support the selective use of retrograde and extracapsular techniques in benign parotidectomy to enhance facial nerve preservation and operative efficiency. In malignant cases, surgical planning should prioritize oncologic and reconstructive goals over specific approaches to dissection. Prospective, multicenter studies using standardized operative protocols and patient‐reported outcomes are needed to further guide surgical decision‐making.

## Author Contributions

**Andrew D. P. Prince**: design, conduct, analysis, drafting, editing; **Christian Schaaff**: design, conduct, analysis, drafting, presenting; **David Forner**: design, conduct, analysis, drafting, editing; **Kimberly Oslin**: design, conduct, analysis, drafting, editing; **Josh Smith**: design, conduct, analysis, drafting, editing; **Michael M. Alevato**: design, conduct, analysis, drafting, editing; **Emma Hershey**: design, conduct, analysis, drafting, editing; **Lisa Chionis**: design, conduct, analysis, drafting, editing; **Mark E. P. Prince**: design, conduct, analysis, drafting, editing; **Steven B. Chinn**: design, conduct, analysis, drafting, editing.

## Disclosures

### Competing interests

None.

### Funding source

None.

## Supporting information

Supplemental Figure 1: Multivariate Regression Analysis of Operative Times in Benign Tumor Resection.

Supplemental Figure 2: Multivariate Regression Analysis of Operative Times in Malignant Tumor Resection.

Supplemental Tables 1 and 2.
