# Supplementary figures and images for: Perioperative Outcomes in Antegrade, Retrograde, and Extracapsular Approaches to Parotidectomy in Benign and Malignant Neoplasms
Source: Otolaryngol Head Neck Surg. 2026 Jan 8;174(2):438–49. doi: 10.1002/ohn.70112 (PMC12860175; doi:10.1002/ohn.70112)

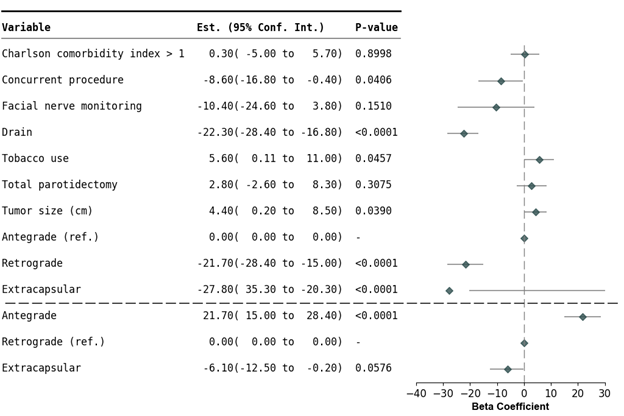

Supplement: Supplementary file 1 — Supplemental Figure 1: Multivariate Regression Analysis of Operative Times in Benign Tumor Resection. [file OHN-174-438-s003.png]

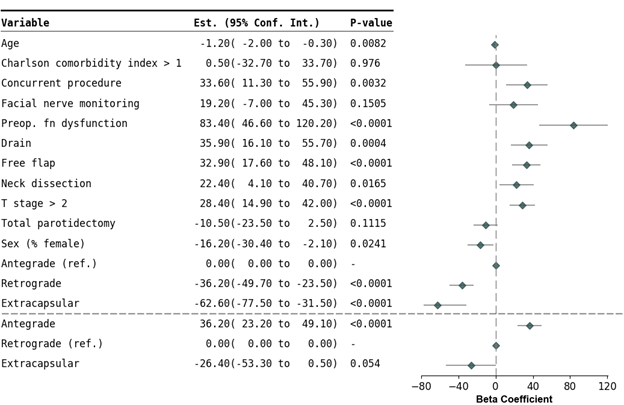

Supplement: Supplementary file 2 — Supplemental Figure 2: Multivariate Regression Analysis of Operative Times in Malignant Tumor Resection. [file OHN-174-438-s001.png]
