# Supplementary material for: Perioperative Outcomes in Antegrade, Retrograde, and Extracapsular Approaches to Parotidectomy in Benign and Malignant Neoplasms
Source: Otolaryngol Head Neck Surg. 2026 Jan 8;174(2):438–49. doi: 10.1002/ohn.70112 (PMC12860175; doi:10.1002/ohn.70112)
Supplement: Supplementary file 3 — Supplemental Tables 1 and 2. [file OHN-174-438-s002.docx]

| **Supplemental Table 1** |  |  |  |  |
| --- | --- | --- | --- | --- |
| Primary vs Metastatic to Parotid Malignant Tumors | Primary | Metastatic | p-Value | Cramer's V |
| Total (n, (%)) | 206 (52) | 188 (48) | - | - |
| Peri-Operative Complication (n, (%)) | 54 (26) | 47 (45) | 0.7828 | 0.01 |
| Hematoma (n, (%)) | 10 (5) | 9 (5) | 0.9752 | 0.00 |
| Seroma (n, (%)) | 11 (5) | 11 (6) | 0.8254 | 0.01 |
| Sialocele (n, (%)) | 2 (1) | 1 (1) | 0.6125 | 0.03 |
| Surgical Site Infection (n, (%)) | 11 (5) | 9 (5) | 0.8122 | 0.01 |
| Skin Flap Necrosis (n, (%)) | 5 (2) | 5 (3) | 0.8836 | 0.01 |
| Salivary Fistula (n, (%)) | 1 (1) | 0 (0) | 0.2543 | 0.05 |
| Stensen Duct Injury (n, (%)) | 0 (0) | 0 (0) | - | - |
| Unintentional Facial Nerve Injury (n, (%)) | 4 (2) | 3 (2) | 0.7337 | 0.01 |
| Purposeful Facial Nerve Resection (n, (%)) | 45 (22) | 34 (18) | 0.3264 | 0.05 |
| Temp. Facial Nerve Weakness (n, (%)) | 64 (40) | 70 (45) | 0.3523 | 0.07 |
| Permanent Facial Nerve Injury (n, (%)) | 17 (11) | 17 (11) | 0.9214 | 0.01 |
| Great Auricular Nerve Injury (n, (%)) | 9 (4) | 6 (3) | 0.5401 | 0.03 |
| Scarring (n, (%)) | 6 (3) | 4 (2) | 0.6192 | 0.02 |
| Frey Syndrome (n, (%)) | 1 (1) | 2 (1) | 0.5067 | 0.03 |
| First Bite Syndrome (n, (%)) | 0 (0) | 1 (1) | 0.2234 | 0.05 |
| Trismus (n, (%)) | 0 (0) | 3 (2) | 0.0346 | 0.09 |
| Other Complications (n, (%)) | 7 (3) | 11 (6) | 0.2423 | 0.06 |
| Tumor Recurrence (n, (%)) | 35 (17) | 49 (26) | 0.0280 | 0.11 |
| Reoperation (n, (%)) | 12 (6) | 12 (6) | 0.8268 | 0.01 |
| Readmission for Surgical Complication (n, (%)) | 4 (2) | 6 (3) | 0.4302 | 0.04 |
| ED Visit for Surgical Complication (n, (%)) | 10 (5) | 7 (4) | 0.5798 | 0.03 |
| Unplanned Clinic Visit for Surgical Complication (n, (%)) | 6 (3) | 5 (3) | 0.8730 | 0.01 |
| Call to Provider for Surgical Complication (n, (%)) | 74 (36) | 71 (38) | 0.7047 | 0.02 |
| Operative Time (min, (sd)) | 287.0 (265.3, 308.7) | 183 (281.6, 327.0) | 0.2788 | - |
| Est. Blood Loss (ml, (sd)) | 149.3 (121.8, 176.8) | 160.4 (131.5, 189.4) | 0.5842 | - |
| Mean Follow-Up Time (months, (sd)) | 18.3 (16.0, 20.7) | 18.6 (16.1, 21.1) | 0.8919 | - |

| **Supplemental Table 2A** |  |  |  |  |  |
| --- | --- | --- | --- | --- | --- |
| Surgeon Specific Analysis: Benign | Total | Antegrade | Retrograde | Extracapsular | p-Value |
| Surgeon 1 (n, (%)) | 1 (<1) | 0 (0) | 0 (0) | 1 (1) | 0.2596 |
| Surgeon 2 (n, (%)) | 64 (18) | 1 (1) | 51 (31) | 12 (13) | <0.0001 |
| Surgeon 3 (n, (%)) | 50 (14) | 3 (3) | 22 (13) | 25 (27) | <0.0001 |
| Surgeon 4 (n, (%)) | 15 (4) | 5 (5) | 3 (2) | 7 (8) | 0.0652 |
| Surgeon 5 (n, (%)) | 1 (<1) | 1 (1) | 0 (0) | 0 (0) | 0.2652 |
| Surgeon 6 (n, (%)) | 34 (10) | 2 (2) | 31 (19) | 1 (1) | <0.0001 |
| Surgeon 7 (n, (%)) | 32 (9) | 7 (7) | 19 (11) | 6 (7) | 0.3467 |
| Surgeon 8 (n, (%)) | 56 (16) | 32 (34) | 12 (7) | 12 (13) | <0.0001 |
| Other Surgeons (n, (%)) | 100 (28) | 43 (46) | 29 (17) | 28 (30) | <0.0001 |
| **Supplemental Table 2B** |  |  |  |  |  |
| Surgeon Specific Analysis: Malignant | Total | Antegrade | Retrograde | Extracapsular | p-Value |
| Surgeon 1 (n, (%)) | 26 (7) | 14 (7) | 11 (7) | 1 (4) | 0.7864 |
| Surgeon 2 (n, (%)) | 36 (9) | 20 (10) | 13 (8) | 3 (12) | 0.6536 |
| Surgeon 3 (n, (%)) | 52 (13) | 20 (10) | 30 (18) | 2 (8) | 0.0703 |
| Surgeon 4 (n, (%)) | 34 (9) | 14 (7) | 18 (11) | 2 (8) | 0.4847 |
| Surgeon 5 (n, (%)) | 85 (22) | 49 (25) | 31 (19) | 5 (19) | 0.2898 |
| Surgeon 6 (n, (%)) | 36 (9) | 14 (7) | 18 (11) | 4 (15) | 0.2924 |
| Surgeon 7 (n, (%)) | 11 (3) | 5 (3) | 3 (2) | 3 (12) | 0.0862 |
| Surgeon 8 (n, (%)) | 43 (11) | 22 (11) | 19 (11) | 2 (8) | 0.8341 |
| Other Surgeons (n, (%)) | 64 (17) | 36 (19) | 24 (14) | 4 (15) | 0.5571 |
